# Making the clock tick: the transcriptional landscape of the plant circadian clock

**DOI:** 10.12688/f1000research.11319.1

**Published:** 2017-06-21

**Authors:** James Ronald, Seth J Davis

**Affiliations:** 1 Department of Biology, University of York, York, YO10 5DD, UK

**Keywords:** Plant circadian clock, Arabidopsis Oscillator, TOC1 promoter

## Abstract

Circadian clocks are molecular timekeepers that synchronise internal physiological processes with the external environment by integrating light and temperature stimuli. As in other eukaryotic organisms, circadian rhythms in plants are largely generated by an array of nuclear transcriptional regulators and associated co-regulators that are arranged into a series of interconnected molecular loops. These transcriptional regulators recruit chromatin-modifying enzymes that adjust the structure of the nucleosome to promote or inhibit DNA accessibility and thus guide transcription rates. In this review, we discuss the recent advances made in understanding the architecture of the *Arabidopsis* oscillator and the chromatin dynamics that regulate the generation of rhythmic patterns of gene expression within the circadian clock.

## Introduction

The daily rotation of the Earth generates predictable diurnal changes in light and temperature. Circadian clocks act as endogenous timekeepers to co-ordinate internal physiological responses to match the predicted environmental condition. The plant circadian clock directly regulates a range of output pathways, which includes hormone signalling, hypocotyl development, metabolism, the floral transition, photosynthesis, and the response to biotic and abiotic stress ^1– 3^. Accordingly, plants with an internal clock that matches the external environment (~24 hours) have enhanced photosynthesis and survival compared to plants with a clock that does not match the external environment ^4^.

Endogenous circadian rhythms are generated through a series of interconnected transcriptional–translational feedback loops, collectively termed the oscillator. Light and temperature signals differentially converge on the plant oscillator through multiple input pathways to provide timing cues in a process termed entrainment ^5, 6^. In plants, light signals at dawn are thought to act as the major entraining signal ^7^. This review will discuss the recent advances made in understanding the transcriptional architecture of the plant oscillator and the chromatin dynamics driving rhythmic gene expression.

## Overview of the oscillator

At the core of the plant oscillator are the morning-expressed, partially redundant MYB domain transcription factors (TFs) CIRCADIAN CLOCK ASSOCIATED 1 (CCA1) and LATE ELONGATED HYPOCOTYL (LHY) ^8– 10^. CCA1/LHY directly antagonise most clock gene expression through binding to the evening element (EE) motif within the promoter ^11– 13^. One target of CCA1/LHY-repressive activity is the *PSEUDO-RESPONSE REGULATOR (PRR) TIMING OF CAB EXPRESSION (TOC1)* ^8, 9, 14^. CCA1/LHY-repressive activity restricts *TOC1* expression to a window around dusk. At dusk, TOC1 accumulates and reciprocally represses *CCA1/LHY* expression in addition to other clock genes ^15, 16^. This mutual antagonism between CCA1/LHY and TOC1 defines the central loop of the *Arabidopsis* oscillator ^7, 9, 17^.

Additional interconnected loops subsequently regulate the activity of the core loop ^7, 18^. At dawn, the TFs TEOSINTE BRANCHED CYCLOIDEA-PCF20/22 (TCP20/22) recruit the co-activator LIGHT REGULATED WD 1 (LWD1) to activate *CCA1/LHY* expression ^19^. LWD1 and its homolog LWD2 are also required to activate the expression of *TOC1* and the related *PRR5*, *PRR7*, and *PRR9* ^20^. PRR5, PRR7, and PRR9 directly associate with the CCA1/LHY promoter and repress *CCA1/LHY* expression ^21, 22^. *PRR9*, *PRR7*, and *PRR5* are sequentially expressed, generating a wave of repressive activity. *PRR9* expression starts at dawn, followed by *PRR7* in the late morning and *PRR5* in the afternoon ^22^. This repressive sequence is re-enforced by the CCA1-related MYB TF REVEILLE8 (RVE8) and its associated homologs, RVE6 and RVE4 ^23^. RVE8 binds to the EE within the *PRR5*, *TOC1*, and *EARLY FLOWERING 4* ( *ELF4*) promoter and activates gene expression by recruiting the co-activators NIGHT LIGHT-INDUCIBLE AND CLOCK REGULATED 1/2 (LNK1/LNK2) ^24– 28^. In the evening, the GARP TF LUX ARRYTHMO (LUX) and the unrelated proteins ELF3 and ELF4 associate to form the evening complex (EC) ^29, 30^. The EC represses the morning-expressed *PRR7* and *PRR9* and evening-expressed *GIGANTEA (GI)* and *LUX* ^29– 34^. LUX and ELF3 have also been recently shown to associate with the promoter of *LNK1/2*, highlighting another potential target of the EC ^35^. Together, this interconnected network of activators and repressors drives rhythmic gene expression within the plant oscillator.

## Chromatin dynamics of the circadian clock

The structure of nucleosomes has a fundamental role in regulating gene expression. A nucleosome is a complex of DNA wound around the histone octamer (two H2A-H2B dimers and a H3-H4 tetramer) ^36^. Each histone unit can be post-translationally modified through a suite of chromatin-remodelling enzymes to generate what is collectively called the histone code ^37^. These modifications regulate the accessibility of the DNA through opening or compacting the histone octamer or by providing a binding site for other chromatin-modifying enzymes ^37^. Modifications associated with transcriptional activation include the acetylation of H3 lysine residues (H3Ac) or tri-methylation of H3K4 (H3K4me3), while repressive markers include the tri-methylation of H3K9 (H3K9me3) and H3K27 (H3K27me3) ^38– 40^.

The promoter regions of *CCA1*, *LHY*, *TOC1*, *GI*, *PRR9*, and *LUX* all display diurnal changes in histone modifications. The levels of H3K9Ac, H3K14Ac, H3K56Ac, and H3K4me3 within the gene promoter peak at the time of maximum gene activation ^41– 44^. Conversely, as gene expression declines, there is a reduction in H3Ac and demethylation of H3K4me3 and an increase in H3K36me2, modifications associated with transcriptional repression ^42^. It has also recently been shown that there are global diurnal changes in H3K9Ac, H3K27Ac, and H3S28P in the promoters of genes associated with the circadian clock and sugar signalling ^45^. Additionally, the association of RVE8 to the *TOC1* promoter is associated with hyperacetylation while the association of CCA1 to the *TOC1* promoter correlates with hypoacetylation ^25, 41^. Diurnal post-translational modification of histones thus has a fundamental role in generating the rhythmic patterns of gene expression within the oscillator.

The factors regulating these histone modifications are beginning to be understood. PRR5, PRR7, and PRR9 directly recruit the Groucho/Tup1 co-repressor TOPLESS (TPL) through an ethylene amphiphilic repression (EAR) domain to repress *CCA1/LHY* expression ^46^. TPL belongs to a multi-gene family of co-repressors that recruit the histone deacetylase (HDA)19 and/or the closely related HDA6 to facilitate gene silencing ^47^. Unlike the other PRRs, TOC1 lacks an EAR domain and cannot directly interact with TPL ^46^. The mechanisms mediating TOC1 repression are therefore unknown. Alongside the PRRs, the EC has also recently been shown to interact with chromatin-remodelling enzymes. ELF3 can co-precipitate with MUT9-like kinase 1–4 (MLK1–4), which promotes the phosphorylation of H3T3 ^48, 49^. H3T3P is associated with heterochromatin formation and gene silencing ^49^. *mlk1–4* single and combination loss-of-function mutants displayed a longer circadian period ^48^. In contrast, loss of function in ELF3, ELF4, or LUX all display circadian arrhythmicity ^50– 53^. Thus, the EC may recruit other co-repressors to repress gene expression.

Other chromatin-remodelling enzymes have also been associated with the plant oscillator. The histone acetyltransferase (HAT) TAF1 and the HDA HD1 regulates the acetylation and de-acetylation of the *TOC1* and *CCA1* promoter, respectively ^44^. However, TAF1 and HDA1 loss-of-function mutants had only a small effect on *TOC1* and *CCA1* expression. *Arabidopsis* has 12 HATs and 18 HDAs, and within each respective class functional redundancy has been observed ^54– 56^. HATs and HDAs are therefore likely to be acting redundantly within the clock. Alongside HATs and HDAs, histone methylases and demethylases have also been implicated within the clock. The H3K4me3 methylase SET DOMAIN GROUP 2 (SDG2/ATRX3) aides clock gene expression and the ability of TOC1 to associate with DNA ^42^. The histone demethylase JUMONJI DOMAIN CONTAINING 5 (JMJD5, also referred to as JMJ30) displays diurnal regulation with expression peaking in the evening ^57^. JMJD5 mutants have a shortening of circadian period, suggesting that JMJD5 has a regulatory role within the oscillator ^57, 58^. Remarkably, *Arabidopsis* JMJD5 has retained conserved functional activity with its human orthologue, which functions within the mammalian clock ^57^. However, the mammalian JMJD5 lacks canonical demethylase activity ^59^. Further work is needed to understand the functional activity of JMJD5 and its role within the *Arabidopsis* clock. It has also been recently shown that 17 different chromatin-remodelling enzymes display diurnal patterns of expression ^60^, further intertwining the relationship between the clock and chromatin remodellers. In summary, the concerted activities of a broad range of histone-modifying enzymes are required within the clock to facilitate the transcriptional regulatory activity of the plant oscillator.

## Conclusions and perspectives

In recent years, much progress has been made in connecting the individual components of the oscillator into an interconnected transcriptional network. However, many questions still persist over the mechanisms of transcriptional regulation. The association of RVE8 to the *TOC1* promoter correlates with hyperacetylation, but neither RVE8 nor LNK1/2 have domains that could recruit HAT directly ^25, 27^. The repressive mechanisms of the core components CCA1/LHY and TOC1 are also poorly understood. TOC1 has been recently shown to co-occupy *PHYTOCHROME INTERACTING FACTOR 3* ( *PIF3*) target promoters and inhibit PIF3-mediated gene activation ^61^. However, whether this is achieved by passively inhibiting HAT recruitment or by actively recruiting co-repressors through an unidentified repression domain is unknown. It also remains unclear whether CCA1/LHY repress gene expression passively or actively, with both mechanisms being proposed in a temporal-dependent manner ^13, 41^. Furthermore, CCA1 and LHY are often grouped together and viewed as a joint operator within the clock. However, CCA1 and LHY have been shown to have distinct roles within the clock ^17, 62^. Future work could investigate the extent of functional overlap between CCA1/LHY.

One noticeable shortage in the plant clock when compared to the mammalian or fungal circadian clock are transcriptional activators ^63, 64^. CCA1/LHY, TOC1, and the plant-specific protein GI were all proposed to act as transcriptional activators within the oscillator ^14, 21, 65^. However, these have now been shown to be an indirect relationship or an effect caused by the mutant background used ^12, 13, 18, 66^. In eukaryotes, the default state of gene expression is often one of a repressive nature ^67^, so transcriptional activators would be expected within the oscillator.

The discovery of the RVE8/LNKs ^25, 27^ and the TCP/LWD complex ^19^ has provided some answers to the mechanisms of transcriptional activation within the oscillator. However, recent mathematical modelling of the oscillator that incorporated RVE8 has shown a non-reliance of the oscillator on transcriptional activation ^18^. The activation of the oscillator genes could be sourced externally. The transcript induction of *CCA1*, *LHY*, *GI*, *PRR9*, *PRR7*, *LNK1*, *LNK2*, *ELF3*, and *ELF4* are all positively regulated by light ^68– 72^. Additionally, the expression of *LUX*, *PRR7*, and *PRR9* is activated in a temperature-dependent manner ^73, 74^. Thus, external environmental signals may participate in gene activation within the clock, while the repressive circuitry of the clock acts to antagonise and attenuate these external gene activation pathways. What is notable in this is the finding that a large proportion of transcription factors are rhythmic and a subset of those can modulate clock parameters ^75^. Together, it appears that known activators within the clock act to fine-tune prevailing environmental antagonism as a form of signal integration.

Transcriptional regulators and the associated chromatin landscape governing transcriptional regulation are only one level nestled within a multi-layered regulatory network. Post-translational modifications, nucleocytoplasmic partitioning, RNA splicing, and protein degradation all have their own essential role in aiding rhythm generation ^76– 78^. It is only through the integration of all of these layers of activity that the plant clock can generate and sustain robust rhythms and facilitate the response to diurnal changes in the environment.
